# Peroxiredoxin‐4, a marker of systemic oxidative stress, is associated with incident heart failure

**DOI:** 10.1002/ejhf.3653

**Published:** 2025-04-06

**Authors:** Navin Suthahar, Sanne G.J. Mourmans, Anouk Achten, Joseph Pierre Aboumsallem, Wouter C. Meijers, Nils Bomer, Isabella Kardys, Ron T. Gansevoort, Stephan J.L. Bakker, Jerremy Weerts, Etto C. Eringa, Kevin Damman, Vanessa van Empel, Rudolf A. de Boer

**Affiliations:** ^1^ Department of Cardiology Erasmus MC, Cardiovascular Institute, Thorax Center Rotterdam The Netherlands; ^2^ Department of Cardiology, CARIM School for Cardiovascular Diseases Maastricht University Medical Center Maastricht The Netherlands; ^3^ Department of Cardiology University of Groningen, University Medical Center Groningen Groningen The Netherlands; ^4^ Division of Nephrology, Department of Internal Medicine University of Groningen, University Medical Center Groningen Groningen The Netherlands; ^5^ Department of Physiology, Amsterdam Cardiovascular Sciences Amsterdam University Medical Center Amsterdam The Netherlands; ^6^ Department of Physiology, CARIM School for Cardiovascular Diseases Maastricht University Maastricht The Netherlands

**Keywords:** Antioxidant enzyme, Biomarker, General population, Heart failure, Oxidative stress, Peroxiredoxin‐4

## Abstract

**Aims:**

Oxidative stress is known to be involved in the pathophysiology of heart failure (HF). To assess oxidative stress, direct quantification of reactive oxygen species would be ideal but this is not feasible due to their short half‐lives. Antioxidant enzymes such as peroxiredoxins, produced as a direct response to oxidative stress, mirror the process and can be more easily quantified. The aim of this study was to examine whether circulating peroxiredoxin‐4 (Prx4), a marker of systemic oxidative stress, associates with incident HF and its subtypes.

**Methods and results:**

We included a total of 8199 individuals from the Prevention of REnal and Vascular End‐stage Disease (PREVEND) community‐based cohort (mean age: 49.8 years; 50.1% women). During a median follow‐up of 12.6 years, 349 (4.3%) HF events occurred of which 118 (33.8%) had HF with preserved ejection fraction. In a Cox proportional hazards model adjusting for age, sex, smoking, diabetes, hypertension, obesity, total and high‐density lipoprotein cholesterol, cholesterol‐lowering medication and renal disease, Prx4 was significantly associated with incident HF (hazard ratio [HR] per 1 standard deviation increase in log‐Prx4: 1.22; 95% confidence interval [CI] 1.09–1.36; *p* < 0.001). Among HF subtypes, Prx4 remained associated with incident HF with preserved (HR 1.27; 95% CI 1.05–1.53) as well as reduced ejection fraction (HR 1.19; 95% CI 1.04–1.37), with no significant difference between the subtypes (*p* = 0.64).

**Conclusion:**

Circulating Prx4 associates with the risk of developing HF, both with preserved and reduced ejection fraction. Future studies should examine whether Prx4 can serve as a real‐time marker of oxidative stress status.

## Introduction

Heart failure (HF) is a heterogeneous clinical syndrome with a complex pathophysiology. It is routinely classified according to left ventricular ejection fraction (LVEF) as HF with preserved (HFpEF) or reduced ejection fraction (HFrEF).^1^, ^2^ Systemic processes such as oxidative stress and inflammation contribute to HF development,^3^, ^4^ and are considered central to the pathophysiology of HFpEF.^1^, ^5^, ^6^ This is in contrast to HFrEF, where although oxidative stress and inflammation are involved in disease progression, the central pathophysiology is reduction in LVEF, most often caused by ischaemic heart disease.^6^, ^7^, ^8^

While several clinical studies have examined the associations between inflammation and the development and progression of HF,^9^, ^10^, ^11^ there is a lack of studies examining associations of oxidative stress in the HF setting. This is because, in population‐based studies, it is difficult to directly quantify the amount of oxidative stress from blood samples as reactive oxygen species (ROS) have short half‐lives, usually in the range of 10^−6^ to 10^−3^ s.^12^

Measuring antioxidant enzyme levels can be a more practical approach, as they are more stable than ROS, and serve as the first line of defence against increased oxidative stress – essentially mirroring the process.^13^, ^14^, ^15^ However, their quantification depends on their presence in the circulation,^12^ as antioxidant enzymes not secreted into the bloodstream cannot be readily detected. Peroxiredoxin‐4 (Prx4)^16^, ^17^ is an antioxidant enzyme that scavenges ROS in the extracellular space and can be measured in the circulation.^18^

Previous reports have indicated that higher circulating Prx4 levels are associated with an increased risk of developing type 2 diabetes, myocardial infarction, and stroke in the PREVEND (Prevention of Renal and Vascular End‐Stage Disease) cohort.^19^, ^20^ However, we were unable to find any large community‐based studies focusing on the relationship between oxidative stress and incident HF.

The objective of the current study was therefore to examine associations of Prx4 (reflecting systemic oxidative stress) with incident HF and its subtypes in the general population. We hypothesized that circulating Prx4 will associate with the risk of developing overall HF, and this may be driven by its strong association with incident HFpEF.

## Methods

### General population cohort

The PREVEND study is a general population cohort from the Netherlands; details on study participants have been previously described.^21^ From the baseline sample (*n* = 8592), we excluded participants with prevalent HF (*n* = 23), missing Prx4 measurements (*n* = 369), and extreme Prx4 outliers (>5 standard deviations above the log‐transformed mean; *n* = 1), leaving 8199 participants available for analysis. The PREVEND study was approved by the local medical ethics committee of the University Medical Center Groningen (approval number: MEC96/01/022) and conformed to the principles drafted in the Helsinki Declaration. Informed consent was provided by all participants. Details on clinical covariates are provided in the online supplementary material.

Follow‐up duration was calculated as the period between the baseline screening visit (1997–1998) and the last contact date, death or 31 December 2010, whichever came first. Patient files were checked in two main hospitals covering the region of Groningen. Individuals suspected of having HF were identified according to the 2012 European Society of Cardiology (ESC) guidelines.^22^ Adjudication of HF events was performed using established protocols.^23^, ^24^ Based on LVEF cutpoint of 50%, HF was subcategorized into HFrEF and HFpEF. Further details can be found elsewhere.^23^, ^24^

### Peroxiredoxin‐4 measurement

Serum Prx4 was measured using a sandwich immunoluminometric assay (ILMA).^20^, ^25^ Limit of blank (LoB) was 0.34 U/L.^25^ The functional assay sensitivity and limit of detection was 0.51 U/L (inter‐assay coefficient of variation [CV] < 20%). The intra‐assay CV was <8% throughout the range of Prx4 levels.

### Statistical analyses

Continuous variables are presented as means (standard deviations) or medians (25th–50th percentile), and categorical variables as number (%). Continuous variables were compared using ANOVA or Kruskal–Wallis test as appropriate, and categorical variables using Pearson's chi‐squared test. Cox proportional hazards models were used to evaluate associations of Prx4 with incident HF and its subtypes. Prx4 was modelled as a continuous and a categorical variable. In continuous models, a value of 0.17 U/L was assigned for Prx4 concentrations less than LoB. Details on Prx4 categorization can be found in the online supplementary material. Model 1 was adjusted for age and sex; Model 2 was additionally adjusted for potential risk factors/confounders (smoking categories, diabetes, hypertension, body mass index categories, total and high‐density cholesterol, cholesterol‐lowering medication usage, and renal dysfunction). In Model 3. potential intermediating conditions (i.e. history of myocardial infarction or stroke) were added to Model 2.^20^ We present results from Model 2 as main results as they are not over‐adjusted by potential intermediate factors.

## Results

**Figure 1:**
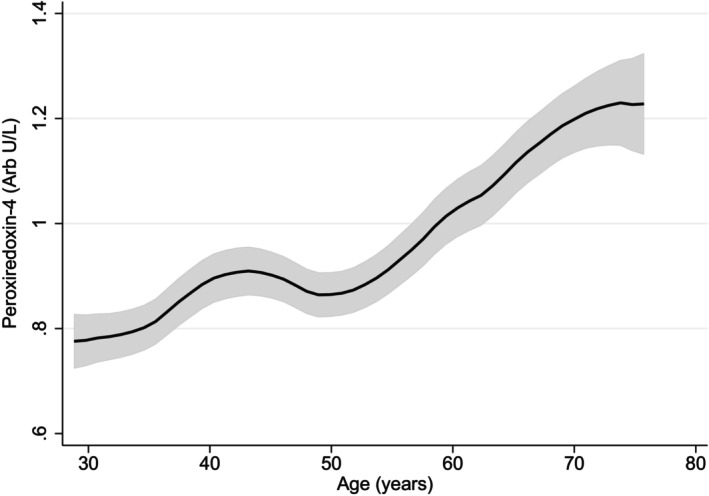
Peroxiredoxin‐4 levels in the general population and associations with age. Associations of peroxiredoxin‐4 with age were modelled using kernel‐weighted local polynomial smoothing technique.

From the PREVEND cohort, we included 8199 individuals without prevalent HF (mean age 49.8 ± 12.6 years, 50.1% female). Median Prx4 level was 0.69 (0.44–1.12) U/L and Prx4 levels increased with age (*Figure* *1*). PREVEND participant characteristics according to Prx4 categories are presented in *Table* *1*.

**Table 1:** PREVEND participant characteristics according to peroxiredoxin‐4 categories

	<0.37 aU/L (*n* = 1665)	0.37–0.51 aU/L (*n* = 964)	0.51–1.12 aU/L (*n* = 3516)	>1.12 aU/L (*n* = 2054)	*p*‐value
Age, years	47.3 (11.7)	48.1 (12.2)	49.5 (12.5)	52.9 (13.1)	<0.001
Female sex, *n* (%)	878 (52.7)	524 (54.4)	1746 (49.7)	965 (47.0)	<0.001
Smoking, *n* (%)					<0.001
Non smokers	433 (26.1)	307 (32.0)	1062 (30.3)	615 (30.1)	
Current smokers	685 (41.3)	347 (36.1)	1147 (32.7)	604 (29.5)	
Quit <1 year	81 (4.9)	33 (3.4)	113 (3.2)	80 (3.9)	
Quit >1 year	461 (27.8)	273 (28.4)	1183 (33.8)	746 (36.5)	
BMI categories, *n* (%)					<0.001
<25 kg/m^2^	876 (53.2)	477 (49.9)	1487 (42.7)	705 (34.8)	
25–30 kg/m^2^	607 (36.9)	354 (37.0)	1429 (41.1)	908 (44.8)	
≥30 kg/m^2^	164 (10.0)	125 (13.1)	564 (16.2)	415 (20.5)	
Diabetes, *n* (%)	20 (1.2)	25 (2.6)	131 (3.8)	127 (6.2)	<0.001
Hypertension, *n* (%)	407 (24.5)	260 (27.1)	1159 (33.1)	934 (45.7)	<0.001
Cholesterol, mmol/L	5.5 (4.9–6.3)	5.5 (4.8–6.3)	5.5 (4.8–6.3)	5.6 (4.9–6.4)	0.051
HDL cholesterol, mmol/L	1.34 (1.09–1.63)	1.31 (1.06–1.60)	1.26 (1.02–1.55)	1.21 (0.99–1.49)	<0.001
Cholesterol lowering medication, *n* (%)	81 (4.9)	53 (5.5)	224 (6.4)	165 (8.1)	<0.001
Renal dysfunction, *n* (%)	24 (1.4)	18 (1.9)	95 (2.7)	149 (7.3)	<0.001
Prevalent CVD, *n* (%)	65 (3.9)	59 (6.1)	226 (6.4)	190 (9.3)	<0.001
UAE, mg/24 h	8.8 (6.2–14.1)	8.6 (6.1–14.7)	9.4 (6.3–16.9)	11.1 (6.8–26.7)	<0.001
NT‐proBNP, ng/L	34.5 (15.7–65.1)	36.1 (16.8–66.4)	36.7 (16.2–71.3)	42.9 (19.1–92.6)	<0.001

Continuous variables are presented as means (standard deviations) or medians (25th–50th percentile), and categorical variables as *n* (%).

BMI, body mass index; CVD, cardiovascular disease; HDL, high‐density lipoprotein; LoB, limit of blank; LoD, limit of detection; NT‐proBNP, N‐terminal pro‐B‐type natriuretic peptide; Prx4, peroxiredoxin‐4; UAE, urinary albumin excretion.

Continuous variables were compared using ANOVA or Kruskal–Wallis test as appropriate, and categorical variables using Pearson's chi‐squared test. The LoB for Prx4 was 0.37 aU/L, LoD was 0.51 aU/L and the 75th percentile was 1.12 aU/L. Prx4 was grouped into four categories (<LoB, between LoB and LoD, between LoD and 75th percentile and ≥75th percentile).

**Figure 2:**
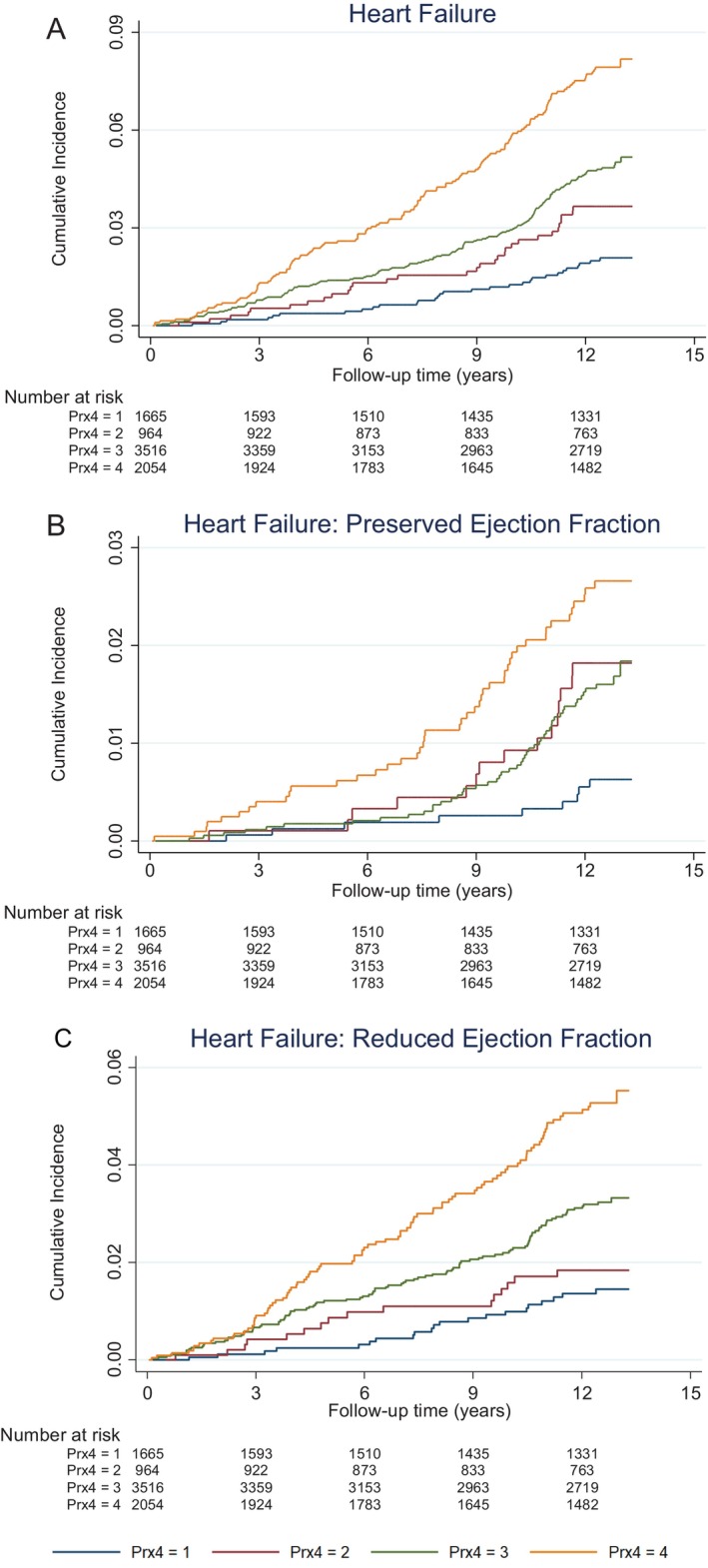
(*A*) Associations of peroxiredoxin‐4 (Prx4) with incident heart failure. (*B*) Associations of Prx4 with incident heart failure with preserved ejection fraction and incident heart failure with reduced ejection fraction. Prx4 was grouped into four categories. Category 1 corresponds to Prx4 < level of blank (LoB) (0.37 aU/L). Category 2 corresponds to Prx4 between LoB (0.37 aU/L) and limit of detection (LoD) (0.51 aU/L). Category 3 corresponds to Prx4 between LoD (0.51 aU/L) and 75th percentile (1.12 aU/L). Category 4 corresponds to Prx4 ≥75th percentile (1.12 aU/L). [Correction added on 8 May 2025, after first online publication: *Figure 2C* was corrected in this version.]

During a median follow‐up of 12.6 (12.24–12.88) years, 349 new‐onset HF events (4.3%) were recorded, of which 118 were HFpEF (33.8%) and 231 were HFrEF (66.2%). The cumulative incidence of HF and its subtypes according to Prx4 categories are shown in *Figure* *2*.

Results from Cox regression models are shown in *Table* *2*. Specifically, after adjustment for potential risk factors and confounders (Model 2), continuous Prx4 was significantly associated with incident HF (hazard ratio [HR] per 1 standard deviation increase in log Prx4: 1.22; 95% confidence interval [CI] 1.09–1.36). Among HF subtypes, Prx4 remained associated with incident HFpEF (HR 1.27; 95% CI 1.05–1.53) and incident HFrEF (HR 1.19; 95% CI 1.04–1.37), with no significant difference between subtypes (*p* = 0.64). When models were additionally adjusted for prevalent cardiovascular disease, effect sizes were slightly attenuated but trends remained similar (*Table* *2*). Results were also broadly similar when Prx4 was handled as a categorical variable (*Table* *2*). When we defined HFpEF as LVEF >40% (*n* = 128), results did not materially change (online supplementary *Table* *S1*).

**Table 2:** Associations of peroxiredoxin‐4 with incident heart failure and its subtypes in the general population

	Model 1		Model 2		Model 3	
	Hazard ratio (95% CI)	*p*‐value	Hazard ratio (95% CI)	*p*‐value	Hazard ratio (95% CI)	*p*‐value
Heart failure						
Continuous Prx4	1.29 (1.16–1.44)	<0.001	1.22 (1.09–1.36)	<0.001	1.19 (1.06–1.33)	0.003
Categorical Prx4						
≤0.37 aU/L	Ref	–	Ref	–	Ref	–
0.37–0.51 aU/L	1.56 (0.94–2.58)	0.083	1.57 (0.95–2.62)	0.079	1.37 (0.82–2.29)	0.223
0.51–1.12 aU/L	1.84 (1.24–2.74)	0.002	1.76 (1.18–2.63)	0.006	1.63 (1.09–2.45)	0.017
≥1.12 aU/L	2.33 (1.56–3.47)	<0.001	2.02 (1.34–3.05)	0.001	1.83 (1.21–2.77)	0.004
Trend across categories	1.30 (1.15–1.46)	<0.001	1.22 (1.09–1.38)	0.001	1.20 (1.06–1.35)	0.003
HFpEF						
Continuous Prx4	1.33 (1.09–1.58)	0.003	1.27 (1.05–1.53)	0.014	1.24 (1.03–1.50)	0.026
Categorical Prx4						
≤0.37 aU/L	Ref	–	Ref	–	Ref	–
0.37–0.51 aU/L	2.64 (1.15–6.03)	0.022	2.81 (1.19–6.64)	0.019	2.57 (1.08–6.08)	0.033
0.51–1.12 aU/L	2.22 (1.09–4.53)	0.028	2.26 (1.06–4.82)	0.035	2.14 (1.00–4.56)	0.050
≥1.12 aU/L	2.77 (1.35–5.70)	0.006	2.66 (1.24–5.73)	0.012	2.48 (1.15–5.34)	0.021
Trend across categories	1.28 (1.05–1.55)	0.013	1.24 (1.01–1.51)	0.036	1.22 (1.00–1.49)	0.056
HFrEF						
Continuous Prx4	1.28 (1.12–1.46)	<0.001	1.19 (1.04–1.37)	0.012	1.16 (1.01–1.33)	0.038
Categorical Prx4						
≤0.37 aU/L	Ref	–	Ref	–	Ref	–
0.37–0.51 aU/L	1.12 (0.59–2.15)	0.727	1.11 (0.58–2.12)	0.762	0.94 (0.49–1.80)	0.850
0.51–1.12 aU/L	1.69 (1.05–2.71)	0.030	1.57 (0.97–2.53)	0.065	1.45 (0.90–2.33)	0.130
≥1.12 aU/L	2.14 (1.32–3.45)	0.002	1.77 (1.09–2.88)	0.022	1.58 (0.97–2.58)	0.068
Trend across categories	1.30 (1.13–1.51)	<0.001	1.21 (1.05–1.41)	0.009	1.19 (1.02–1.38)	0.023

Continuous Prx4 was log‐transformed and standardized, and hazard ratios in continuous models should be interpreted per standard deviation change in log‐transformed Prx4. The LoB for Prx4 was 0.37 aU/L, LoD was 0.51 aU/L, and the 75th percentile was 1.12 aU/L. In categorical analyses, Prx4 was grouped into four categories (<LoB, between LoB and LoD, between LoD and 75th percentile and ≥75th percentile). Prx4 < LoB was taken as the referent category. Model 1 is adjusted for age and sex. Model 2 = Model 1 + smoking, diabetes, hypertension, body mass index categories, total cholesterol, high‐density cholesterol, cholesterol‐lowering medication, chronic kidney disease. Model 3 = Model 2 + history of cardiovascular disease.

CI, confidence interval; HFpEF, heart failure with preserved ejection fraction; HFrEF, heart failure with reduced ejection fraction; LoB, limit of blank; LoD, limit of detection; Prx4, peroxiredoxin‐4.

## Discussion

In the current study, we examined, for the first time, the relationship between circulating antioxidant enzyme Prx4 and the risk of developing HF and its subtypes. We hypothesized that Prx4 would be associated with HF risk, with a potentially stronger association with incident HFpEF than HFrEF. While our findings confirmed a strong association between Prx4 and HF risk, this association did not differ among HF subtypes. Overall, our results align with experimental studies indicating that oxidative stress contributes to the pathophysiology of both HFpEF and HFrEF.^3^, ^26^, ^27^

A few previous studies examined associations between oxidative stress and adverse clinical outcomes in HF patients using more passive markers of oxidative stress, such as serum free thiols. As these markers reflect the total antioxidant capacity of the extracellular fluid (i.e. passive buffering ability against oxidative damage), lower levels of serum free thiols were associated with a higher risk of adverse clinical outcomes.^28^ While serum free thiols have also been investigated in the general population in relation to incident myocardial infarction and stroke (with low levels correlating with high cardiovascular risk),^29^ they have not been studied in relation to incident HF.

In the current study, we quantified oxidative stress using the antioxidant enzyme Prx4, which reflects a more active component of oxidative stress response. Indeed, peroxiredoxins act as ‘first responders’ to oxidative stress, even at very low H_2_O_2_ levels, and genetic disruption of peroxiredoxins increases the susceptibility of cells to oxidative stress.^30^ However, most peroxiredoxins function intracellularly, making it difficult to detect them in the circulation – except for Prx4, which is actively secreted.^18^ We found that higher circulating Prx4, indicative of increased systemic oxidative stress, was strongly associated with the risk of developing HF, including both HFpEF and HFrEF. These findings suggest that oxidative stress plays an equally important role in HF development, regardless of ejection fraction.

Although this is the first study to examine the relationship between systemic oxidative stress and incident HF and its subtypes, several limitations deserve consideration. First, it remains unclear whether Prx4 is primarily produced to counteract intracellular oxidative stress (and a fraction of this is secreted into the extracellular space) or whether its primary function is to counteract oxidative stress in the extracellular space.^31^ Second, as Prx4 was measured only at a single time point, it only captures a ‘snapshot’ of oxidative stress status. Future studies with repeated Prx4 over time could provide better insight into how temporal changes in oxidative stress relate to the risk of developing HF and its subtypes. Third, in the current study we used Prx4 concentrations (i.e. quantity) as the sole marker of oxidative stress. Previous studies have shown that antioxidant activity of enzymes such as glutathione peroxidase 3, superoxide dismutase and catalase can also be measured in the serum (or in erythrocytes), and enzyme activity inversely correlates with cardiovascular risk.^32^, ^33^ Future studies should therefore examine the relationship between circulating antioxidant enzyme levels (i.e. quantity) as well as function (i.e. activity) with incident HF and its subtypes. Fourth, the PREVEND study oversampled individuals with mildly elevated urinary albumin excretion (≥10 mg/L), potentially biasing the sample toward higher baseline cardiovascular risk. However, this is unlikely to affect the interpretation of results, as previous research has demonstrated that findings from the PREVEND study align well with those from other general population cohorts.^9^ Furthermore, there was no evidence of an interaction between urinary albumin excretion category and the association between Prx4 and incident HF (*p* for interaction = 0.61). Fifth, this study was conducted on a predominantly White population necessitating validation of our findings in other ethnicities/population groups. Finally, this study only establishes an association between elevated Prx4 levels and HF but does not prove causation. Experimental studies are needed to determine whether Prx4 is merely a biomarker of oxidative stress or plays a more active role in HF pathophysiology.

## Conclusion

Higher circulating Prx4 levels among community‐dwelling adults are associated with the risk of developing HF with preserved as well as reduced ejection fraction. Future studies should examine whether Prx4 can serve as a real‐time marker of oxidative stress status.

## Supporting information


**Appendix S1.** Supporting Information.
