# Supplementary material for: Peroxiredoxin‐4, a marker of systemic oxidative stress, is associated with incident heart failure
Source: Eur J Heart Fail. 2025 Apr 6;27(5):905–11. doi: 10.1002/ejhf.3653 (PMC12103958; doi:10.1002/ejhf.3653)
Supplement: Supplementary file 1 — Appendix S1. Supporting Information. [file EJHF-27-905-s001.docx]

**SUPPLEMENTARY METHODS**

Study participants underwent a comprehensive medical history, physical examination and phlebotomy. Blood samples were collected after an overnight fast in all participants, and stored at -80 degree centigrade for future analysis. Based on smoking status participants were classified as non-smokers, active smokers, stopped smoking < 1 year and stopped smoking ≥ 1 year. Body mass index (BMI) was calculated as weight/height^2^ (kg/m^2^). Overweight was defined as BMI between 25 and 30 kg/m^2^ and obesity was defined as BMI ≥ 30 kg/m^2^. Blood pressure (BP) was calculated as the average of 2 seated measurements. Hypertension was defined as systolic BP ≥ 140 mmHg, diastolic BP ≥ 90 mmHg, or the use of antihypertensive medication. Diabetes was defined as a fasting glucose ≥ 7.0 mmol/L (126 mg/dL), a non-fasting glucose ≥ 11.1 mmol/L (200 mg/dL), or the use of anti-diabetic medication. Estimation of the glomerular filtration rate (eGFR) was done using the chronic kidney disease epidemiology collaboration (CKD-EPI) equation based on creatinine and cystatin C levels in the majority of the population (n=7752). In individuals with unavailable cystatin-C measurements (n=457), eGFR was computed using the CKD-EPI equation based on creatinine levels. Renal dysfunction was defined as eGFR < 60 ml/min/1.73m^2^. History of CV disease (ie, myocardial infarction and stroke) were based on individuals’ medical history derived from a structured questionnaire i.e., hospitalization ≥ 3 days as a result of this condition; this was complemented by a review of the medical report. In analyses on PREVEND cohort.

The limit of blank (LoB) for Peroxiredoxin-4 (Prx4) in the PREVEND cohort was 0.34 U/L; the functional assay sensitivity and limit of detection (LoD) was 0.51 U/L; and the 75^th^ percentile was 1.12 U/L. Cox proportional hazards models were used to evaluate associations of Prx4 with incident HF and its subtypes. Prx4 was modelled as a continuous and a categorical variable. In continuous models, a value of 0.17 U/L (i.e., LoB/2) was assigned for Prx4 concentrations less than LoB. In categorical models, Peroxiredoxin-4 (Prx4) was grouped into 4 categories. Category 1 corresponds to Prx4 levels less than the LoB (0.37 U/L). Category 2 corresponds to Prx4 levels between LoB (0.37 U/L) and LoD (0.51 U/L). Category 3 corresponds to Prx4 levels between LoD (0.51 U/L) and the 75^th^ percentile (1.12 U/L). Category 4 corresponds to Prx4 greater than or equal to the 75^th^ percentile (1.12 U/L).

**Table S1.** Associations of Peroxiredoxin-4 with Incident Heart Failure with Preserved Ejection Fraction (LVEF > 40%)

|  | Model 1 | | Model 2 | | Model 3 | |
| --- | --- | --- | --- | --- | --- | --- |
|  | **Hazard Ratio (95% CI)** | **P-value** | **Hazard Ratio (95% CI)** | **P-value** | **Hazard Ratio (95% CI)** | **P-value** |
| Continuous Prx4 | 1.32 (1.11-1.58) | 0.002 | 1.26 (1.05-1.51) | 0.014 | 1.23 (1.03-1.49) | 0.026 |
| Categorical Prx4 |  |  |  |  |  |  |
| ≤ 0.37 aU/L | Ref | - | Ref | - | Ref | - |
| 0.37-0.51 aU/L | 2.81 (1.24-6.37) | 0.013 | 2.96 (1.27-6.95) | 0.012 | 2.68 (1.14-6.30) | 0.024 |
| 0.51-1.12 aU/L | 2.44 (1.20-4.96) | 0.013 | 2.42 (1.14-5.13) | 0.022 | 2.27 (1.07-4.83) | 0.033 |
| ≥1.12 aU/L | 3.01 (1.47-6.16) | 0.003 | 2.78 (1.30-5.97) | 0.008 | 2.58 (1.20-5.54) | 0.015 |
| Trend across categories | 1.30 (1.08-1.57) | 0.006 | 1.24 (1.02-1.51) | 0.028 | 1.22 (1.00-1.49) | 0.045 |

**Continuous Prx4 was log transformed and standardized, and hazard ratios in continuous models should be interpreted per SD change in log-transformed Prx4.** The limit of blank (LoB) for Prx4 was 0.37 aU/L, limit of detection (LoD) was 0.51 aU/L, and the 75^th^ percentile (P75) was 1.12 aU/L. In categorical analyses, Prx4 was grouped into 4 categories (<LoB, between LoB and LoD, between LoD and 75^th^ percentile and ≥75^th^ percentile). Prx4 < LoB was taken as the referent category. Model 1 is adjusted for age and sex. Model 2 = Model 1 + smoking, diabetes, hypertension, body mass index categories, total cholesterol, high-density cholesterol, cholesterol lowering medication, chronic kidney disease. Model 3 = Model 2 + history of cardiovascular disease.
